# 00002 Translational Fellows as a mechanism to improve throughput of university technology commercialization

**DOI:** 10.1017/cts.2021.515

**Published:** 2021-03-30

**Authors:** Everett G. Hall, Tom M. Krenning, Michael S. Kinch

**Affiliations:** Washington University in St. Louis

## Abstract

ABSTRACT IMPACT: This work aims to identify best practices for university-based asset development programs to improve commercialization throughput, which in turn will drive innovation in the biomedical space and directly contribute to improved human health. OBJECTIVES/GOALS: University technology transfer exhibits a high rate of failure, often due to a lack of researcher experience or early-stage financial capital. The LEAP program at Washington University (WUSTL) was created to address these needs. The goal of this study is to assess the performance of LEAP against similar gap funds and further improve program operations. METHODS/STUDY POPULATION: The goals of LEAP are achieved by providing university inventors with individualized consulting and feedback from industry experts, as well as awarding funding to the most promising projects. To determine whether these activities are impactful, we distributed an awardee report form to collect data on all funded LEAP projects, and then combined the results with project registration information. We also collected records Office of Technology Management, including invention disclosures, licenses, and startup creations. The resulting dataset was used to calculate program metrics and then evaluated against comparable gap funds. Sentiment data from participant surveys were also analyzed to assess perceived program value and knowledge transfer. RESULTS/ANTICIPATED RESULTS: As of the Sp2020 cycle, LEAP has funded 76 projects. Resubmitted projects had a funding rate of 52%, vs. 34% for new projects. Of the startups founded off of WUSTL intellectual property since 2016, nearly two-thirds had previously participated in LEAP. Funded LEAP projects also had a 29% licensing rate, which is comparable to similar gap funds. Lastly, participants self-reported an increase in knowledge across a range of commercialization areas. DISCUSSION/SIGNIFICANCE OF FINDINGS: The increased repeat funding rate and self-reported knowledge suggest that LEAP is impactful in building commercialization proficiency. The licensing rate and prevalence of LEAP projects in WUSTL startups also indicate that LEAP is indeed promoting tech transfer. Together, these results suggest that LEAP could be a model for other institutions.

